# A method for the madness: An international survey of health professions education authors’ journal choice

**DOI:** 10.1007/s40037-022-00698-9

**Published:** 2022-02-22

**Authors:** Eliot L. Rees, Oliver Burton, Aqua Asif, Kevin W. Eva

**Affiliations:** 1 School of Medicine, Keele University, Keele, UK; 2 Research Department for Primary Care and Population Health, University College London, London, UK; 3 School of Medical Education, Faculty of Medical Sciences, Newcastle University, Newcastle upon Tyne, UK; 4 School of Medicine, University of Leicester, Leicester, UK; 5 Centre for Health Education Scholarship and Department of Medicine, University of British Columbia, Vancouver, BC Canada

**Keywords:** Publishing, Medical education, Survey, Scholarship

## Abstract

**Introduction:**

Scholarship is a key activity in health professions education (HPE). When disseminating scholarly work, how one selects the journal to which they submit is often argued to be a key determinant of subsequent success. To draw more evidence-based recommendations in this regard, we surveyed successful scholars working in HPE regarding their perspectives and experiences with journal selection.

**Methods:**

We conducted an international survey of HPE scholars, investigating their decisions regarding journal choice. Corresponding authors were identified from a sample of 4000 papers published in 2019 and 2020. They were invited via email with up to four reminders. We describe their experience and use principle component and regression analyses to identify factors associated with successful acceptance.

**Results:**

In total, 863 responses were received (24.7% response rate), 691 of which were included in our analyses. Two thirds of respondents had their manuscripts accepted at their first-choice journal with revisions required in 98% of cases. We identified six priority factors when choosing journals. In descending order of importance, they were: fit, impact, editorial reputation, speed of dissemination, breadth of dissemination, and guidance from others. Authors who prioritised fit higher and who selected a journal earlier were more likely to have their manuscripts accepted at their first-choice journal.

**Discussion:**

Based on our results we make three recommendations for authors when writing manuscripts: do not be disheartened by a revise decision, consider journal choice early in the research process, and use the fit between your manuscript and the journal as the main factor driving journal choice.

**Supplementary Information:**

The online version of this article (10.1007/s40037-022-00698-9) contains supplementary material, which is available to authorized users.

## Introduction

Scholarship in Health Professions Education (HPE) is considered one of the key activities of clinician educators [1]. This competency has been defined as the ability to ‘contribute to the development, dissemination, and translation of health professions education knowledge and practices’ [1]. In fact, making one’s efforts publicly available with peer review is a defining feature of scholarship according to Boyer’s classic model [2]. This creates a particular challenge for our field because most come to it through serendipity, with little formal training in scholarship, and with a wide array of backgrounds, interests, and roles [3]. Much advice has been generated in an effort to support individuals who are new to the field to have publication success, but little empirical work is available to help separate good advice from lore. What journals should they choose and why? How should they engage with or rely upon peer review processes? To what extent are publication priorities malleable and what impact might changing perspectives have?

Finding answers to such questions would be particularly timely given increased global awareness of equity, diversity, and inclusion issues. Medical education literature in particular has been dominated by research from North America, the United Kingdom, the Netherlands, and Australia [4, 5]. Other countries are less prominent for a variety of reasons including population size and lack of priority having been given to scholarship in this area. To ensure those countries are not excluded for unacceptable reasons like systemic bias, however, it is important that we determine and share the trustworthy ‘tricks of the trade’ that have been learned by individuals who successfully published a manuscript in this field [6]. To that end, we sought information regarding successful authors’ publication strategies and experiences pertaining to a wide range of issues encountered in HPE scholarship to offer an evidence base and guidance to those who would benefit from understanding the norms in this field.

What priorities do authors hold when selecting where to publish their work? To what extent do those priorities vary between different demographic groups? Multiple factors have been previously identified as important considerations for medical education authors, including impact factor and prestige [7]. Work in health sciences more generally, from the US, has corroborated the primacy of prestige when choosing a journal [8]. If that first choice is unsuccessful, acceptance odds and frequency of the topic appearing in the journal became more important [8]. A more recent study of medical and dental academics in India identified the five most important factors as: the databases in which the journal is indexed, the ability to submit online, impact factor, peer review, and publication fees [9]. While the inclusion of indexing and publication fees here may be a consequence of the rise in both predatory and legitimate open access journals, variability in these rankings across studies may also be the result of contextual differences, thereby suggesting the importance of examining these issues in our specific field.

Of course, authors’ priorities for pursuing a particular journal are not the only determinants of where a work is eventually published. As such, it is important to explore their choices in relation to what happened to the paper with respect to peer review and subsequent revision. While long known to have reliability problems, peer review’s primary value may be highlighting diversity of opinion, helping authors to see ways in which their work might be misinterpreted relative to the way in which it was intended [10]. A study of the prepublication history of 80,748 biological sciences articles across 923 journals supports this argument by showing that articles that were given more challenging feedback (presumably, given that the paper was rejected) ultimately accumulated more citations relative to articles that were accepted at the first journal to which they were submitted [11].

These big picture issues require further exploration if we are to both offer good advice to authors in our field and reflect on the strengths and priorities of the field as a scientific enterprise in its own right. With this study, therefore, we surveyed successful scholars working in HPE regarding their perspectives and experiences with journal selection.

## Methods

### Design

We conducted a cross-sectional online questionnaire study of recently published HPE scholars. Ethical approval was granted by the Keele Institute for Innovation and Teaching Excellence—Educational Research Ethics Committee (KIITE-EREC) at Keele University (Ref: KR-210048).

### Sampling and recruitment

To identify a sample, we generated a list of HPE papers published in 2019 and 2020 by searching PubMed using the query: ‘Education, medical’ OR ‘health professions education’ across all database fields. We did not deliberately exclude non-English articles, but our search strategy would have implicitly focused on the English literature. Letters and editorials were excluded because authors of such pieces do not generally undertake the same choice-making processes regarding where to submit. We then extracted the names and email addresses of the corresponding authors from all remaining papers. Duplicate names were reviewed and the most recent research paper for each unique author was selected. We then randomly selected 4000 unique corresponding authors and invited them, via email, to complete our questionnaire. Four reminders were sent at one-week intervals. For the same reason as expressed above, exclusion criteria applied after survey completion were (a) the author identified the manuscript as a letter or editorial and (b) the author identified the manuscript as having been commissioned.

### Survey development

We developed a novel questionnaire for this study by conducting a literature review aimed at identifying any existing measures that could be adapted to suit our research questions and supplementing that with personal experience to identify important aspects of journal choice. Individual items were generated following guidelines for best practice. This included stating both positive and negative sides in question stems, developing response options that were comprehensive and mutually exclusive, and using balanced scales with construct-specific labels [12]. Questions about demographic and professional background were adapted from a previous study of HPE authors [13]. Items exploring perspectives on peer review feedback were developed based on characteristics of reviewers’ comments identified in a systematic review of tools to assess quality of peer review reports [14]. Draft versions of the questionnaire were discussed amongst the researchers until a final draft was complete.

We then submitted the draft for expert review by inviting a panel of four senior HPE researchers (two male, two female, including clinical academics and scientists) to offer feedback on the questionnaire regarding its clarity and relevance. We used their guidance to revise the survey, rephrasing unclear items and adding response options, and conducted a trial administration with five early career authors to identify any further concerns regarding clarity and to provide an accurate estimate of duration for completion.

The final version of the survey included 59 items divided into 10 sections (see Electronic Supplementary Material [ESM] for the survey). Participants were presented with the title of the manuscript that was used to identify them and they were asked to answer all questions based on that manuscript.

### Data analysis

We used SPSS version 27 (SPSS Inc., Chicago, IL) for all analyses and considered *p*-values less than 0.01 to be statistically significant. We present categorical variables as frequencies with percentages and continuous variables as means with their standard deviation.

To assess for nonresponse bias we conducted a wave analysis [15]. That is, we used late responders (those who participated after the final reminder was sent) as proxies for nonrespondents and compared their responses to immediate responders (those who participated after the initial invitation was sent). In doing so, we calculated a nonresponse statistic for each of the items focused on rating factors’ influence on journal selection (i.e., our primary outcome).

We grouped career stages into trainee (students and clinical trainees), junior academic (below senior lecturer/associate professor), and senior academic (senior lecturer/associate professor and above). We grouped country of corresponding author into those that are most represented in the HPE literature (USA, UK, Canada, Australia, the Netherlands) [4, 5] and others.

To reduce data and create more robust measures of the constructs of interest, we performed principal component analyses (PCA) on the 10 “motivation for publication” items and the 21 “journal priority items” with oblique rotation. The Kaiser-Meyer-Olkin measure verified sampling adequacy for the two analyses at KMO = 0.775 and 0.839 for motivations and priorities, respectively. Subsequent analyses used computed factor scores based on the mean of their components. Missing data were replaced with item means where possible.

Finally, we performed linear and logistic regression analyses to identify associations between author demographics and priorities with submission outcome. We included the stage at which authors decided on a first-choice journal in these models because deciding early and tailoring one’s article to the journal style is commonly advised.

## Results

### Participants

Of the 4000 email invitations, 509 were returned as undeliverable. Of the remaining 3491 individuals, 863 responded to our survey, giving an overall response rate of 24.7%. From this group, we excluded 98 responses focused on ineligible contributions to the literature (19 editorials, 7 letters, and 72 commissioned articles), 3 duplicate responses, and 75 incomplete responses (33 completed the consent form but not the questionnaire and 42 completed the demographic items only). Our final dataset, therefore, consisted of 691 responses, a final response rate of 21.7% (American Association for Public Opinion Research, Standard definition 4) [16]. Wave analysis revealed a median nonresponse bias statistic of 0.03, suggesting very little difference between respondents and proxy nonrespondents.

Respondents were aged 22 to 90 with a mean of 44.8 (SD = 11.2) years. They had a median of 27 (range, 1–700) publications. Of the respondents, 471 (68%) were from the dominant HPE publishing countries (United States, United Kingdom, Canada, Australia, the Netherlands). Further demographics are presented in Table S1, found in ESM.

### Prepublication history

Manuscripts were initially submitted to 233 different first-choice journals. Of the 337 (63.5%) manuscripts that were accepted at the first journal to which they were submitted, 6 (1.8%) were accepted without revisions, 172 (51.0%) were accepted after minor revisions, and 159 (47.2%) were accepted after major revisions. These manuscripts required a mean of 1.4 (SD = 0.7) rounds of revision. The likelihood of being accepted at the first journal to which authors submitted was comparable for those submitting from a “dominant” country (67.9%) relative to a “non-dominant” country (62.4%; Chi-squared = 2.0, *p* = 0.16). The same is true for those whose corresponding authors varied in experience level—trainees, junior academics, and senior academics = 57.1%, 68.8%, and 68.2%, respectively (Chi-squared = 5.9, *p* = 0.05); for those whose research type prioritization differed—quantitative, qualitative, and mixed methods = 66.1%, 71.4%, and 64.9%, respectively (Chi-squared = 1.8, *p* = 0.40); and for male vs female corresponding authors = 61.7% and 70.1%, respectively (Chi-squared = 5.2, *p* = 0.02).

Journals took a mean of 8.4 (SD = 7.8) weeks to make a first decision and overall, the process took a mean of 19.6 (SD = 19.6) weeks between first submission and ultimate acceptance. On average, papers were sent to a total of 1.5 journals before publication was achieved. Of the 194 (36.5%) manuscripts that were not accepted at the first journal, 105 (54.1%) were rejected without peer review and 89 (45.9%) were rejected after review. The modal approach to revising manuscripts before submitting to another journal was to change the formatting to meet the new journal’s requirements; this was the case for those that were rejected after peer review and without peer review but the proportion who fell into that category varied markedly (33.0% of those who received peer review made only formatting changes vs 65.0% of those who were rejected without peer review; Chi-squared = 41.5; [*p* < 0.001]). For those rejected after peer review, 8 (9.1%) tweaked the wording, 24 (27.3%) made changes to reporting without significant changes to content, 22 (25%) made substantial changes to the manuscript, and 5 (5.7%) just resubmitted the original manuscript. When comparing the 38 that made substantial changes to the 179 who changed formatting, wording, or reporting, the average number of journals pursued before publication was achieved was 2.6 and 2.5, respectively (t = 0.4, *p* = 0.68). The average length of time the subsequent journal required to offer a first decision was 6.7 weeks. There was no significant difference in the duration between rejection from first journal and final acceptance between those who made substantial changes (mean = 23.1, SD = 20.8 weeks) and those who made formatting, wording, or reporting changes (mean = 35.2, SD = 39.0) (t = −1.42, *p* = 0.17).

### Motivations for publishing

In order of importance rating (which ranged from 1 to 5), respondents’ motivations for publication were as follows: to communicate with others (mean = 4.26, SD = 0.71), to advance knowledge in the field (mean = 4.16, SD = 0.81), to develop national/international reputation (mean = 3.25, SD = 1.12), to support career development of co-authors (mean = 3.23, SD = 1.27), to get feedback from peer reviewers (mean = 3.08, SD = 1.10), to enable networking with others in the field (mean = 3.08, SD = 1.11), to enable promotion or other type of career advancement (mean = 3.01, SD = 1.30), to enjoy the thrill of seeing work in print (mean = 2.79, SD = 1.16), to assist with winning grants and research support (mean = 2.21, SD = 1.17), and to act as a catalyst for attracting high quality staff and students (mean = 2.21, SD = 1.15) (Table S2 in ESM).

The application of principal component analysis to these motivations identified three factors with eigenvalues greater than 1; they explained 58.9% of the variance (Table S3 in ESM) and the scree plot’s inflexion point further suggested that three factors underlaid the responses to individual questions. Content review of the item clustering suggested that the three factors represent motivation for personal promotion, desire to advance the field’s knowledge, and capacity building.

Multivariate linear regression analyses (Tab. 1) performed using each of these factors as a dependent variable indicated that females, authors from non-dominant countries, and those at earlier career stages were statistically more motivated than their counterparts by personal promotion, but the effect of gender was small as illustrated by the beta weights presented in Tab. 1. Authors from non-dominant countries and at more senior career stages were more motivated by capacity building than their counterparts. There were no differences between genders, country groups, or career stage in the extent to which respondents were motivated to advance knowledge in the field. Those who were published at the first journal to which they submitted were equally likely to be motivated by personal promotion, advancing the field’s knowledge, or capacity building as were other authors.

**Table 1:** Multivariate regression analyses of association between author demographics and motivations for publication

Dependent variables:			Personal promotion	Advancing knowledge	Capacity building
**Independent variables:**	*Gender (Male)*	*B (95% CI)*	*−0.19* *(−0.32, −0.08)*	*n/s*	*n/s*
	Male	Mean (SD)	2.99 (0.80)	4.20 (0.70)	2.53 (0.91)
	Female	Mean (SD)	3.09 (0.79)	4.22 (0.66)	2.50 (0.89)
	*Dominant country*	*B (95% CI)*	*−0.46* *(−0.59, −0.34)*	*n/s*	*−0.27* *(−0.41, −0.12)*
	Dominant country	Mean (SD)	2.91 (0.76)	4.21 (0.69)	2.44 (0.86)
	Other	Mean (SD)	3.34 (0.80)	4.22 (0.65)	2.68 (0.96)
	*Career stage*	*B (95% CI)*	*−0.15* *(−0.22, −0.07)*	*n/s*	* 0.15* *(0.06, 0.24)*
	Trainee	Mean (SD)	3.18 (0.81)	4.13 (0.94)	2.30 (0.89)
	Junior academic	Mean (SD)	3.17 (0.79)	4.17 (0.71)	2.53 (0.91)
	Senior academic	Mean (SD)	2.89 (0.80)	4.28 (0.67)	2.61 (0.89)

*n/s* not significant

### Priorities when selecting a journal

Respondents’ ratings indicated that the most important priorities when selecting a journal in the first instance were match between the journal’s readership and the audience the author hoped to reach (mean = 4.13) along with focus of the journal (4.12). These were followed by familiarity with journal (3.66), manuscript types accepted (3.63), reputation for publishing rigorous research (3.63), database indexing (3.48), and impact factor (3.47) (Table S4 in ESM). In response to a free text question eliciting any other priorities, three other factors were frequently reported: responding to a call for a special issue, having published previous work from the same research programme in the journal, and a requirement to submit to that journal as a condition for abstract acceptance at a conference.

PCA identified six factors with eigenvalues greater than 1 that explained 61.9% of variance and were reinforced by the location of the inflexion point on the scree plot (Table S5 in ESM). Content analysis of the items within each cluster suggested that these factors reflect editorial reputation (reputation of editor, reputation of editorial board, reputation for useful feedback during peer review), fit between manuscript and journal (focus of the journal, match between the journal’s readership and the audience one hoped to reach, manuscript types accepted, familiarity with journal), guidance from others (department head/supervisor, colleagues), speed of dissemination (acceptance rate, ability to publish open access, time taken to publish accepted manuscripts, reputation for making quick decisions), impact (impact factor, attention the journal gets in press, reputation for publishing rigorous research, databases in which journal is indexed), and breadth of journal dissemination (link with a society or organisation, geographic distribution of readership).

Multivariate regression analyses (Tab. 2) indicated that females prioritised guidance from others and fit more than males. Authors from non-dominant HPE publishing countries prioritised speed of dissemination, impact, editorial reputation, and breadth of dissemination more than those from dominant countries. Authors at earlier career stages prioritised guidance from others more than more senior authors.

**Table 2:** Multivariate regression analyses of association between author demographics and priorities for first choice journal

Dependent variables:			Editorial reputation	Fit	Guidance from others	Speed of dissemination	Impact	Breadth of dissemination
**Independent variables:**	*Gender (Male)*	*B (95% CI)*	*n/s*	*−0.14* *(−0.24, −0.05)*	*−0.29 (−0.40, −0.15)*	*n/s*	*n/s*	*n/s*
	Male	Mean (SD)	2.57 (1.09)	3.81 (0.65)	1.95 (0.93)	2.20 (0.90)	3.56 (0.83)	2.23 (0.76)
	Female	Mean (SD)	2.59 (1.00)	3.95 (0.61)	2.23 (1.04)	2.56 (0.91)	3.29 (0.84)	2.20 (0.74)
	*Dominant country*	*B (95% CI)*	*−0.47* *(−0.63, −0.30)*	*n/s*	*n/s*	*−0.54 (−0.69, −0.40)*	*−0.47 (−0.61, −0.34)*	*−0.21 (−0.34, −0.09)*
	Dominant country	Mean (SD)	2.45 (1.01)	3.90 (0.65)	2.04 (0.99)	2.38 (0.84)	3.17 (0.84)	2.15 (0.73)
	Other	Mean (SD)	2.89 (1.07)	3.85 (0.58)	2.18 (1.01)	2.90 (0.94)	3.64 (0.75)	2.36 (0.79)
	*Career stage*	*B (95% CI)*	*n/s*	*n/s*	*−0.50 (−0.59, −0.41)*	*n/s*	*n/s*	*n/s*
	Trainee	Mean (SD)	2.71 (1.08)	3.80 (0.61)	2.72 (1.08)	2.64 (0.95)	3.41 (0.87)	2.24 (0.79)
	Junior academic	Mean (SD)	2.57 (1.06)	3.90 (0.65)	2.23 (1.04)	2.58 (0.92)	3.29 (0.87)	2.24 (0.78)
	Senior academic	Mean (SD)	2.56 (1.02)	3.91 (0.63)	1.71 (0.75)	2.48 (0.87)	3.29 (0.81)	2.19 (0.73)

*n/s* not significant

An additional multivariate logistic regression demonstrated that the extent to which an author prioritised fit yielded the greatest odds of being accepted at the first journal (Exp(B) = 2.05, 95% CI 1.48–2.83) followed by the extent to which they prioritised speed of dissemination (Exp(B) = 1.78, 95% CI 1.39–2.29). Prioritising journal impact reduced the odds of being accepted at the first journal (Exp(B) = 0.38, 95% CI 0.29–0.50) as did deciding later in the manuscript writing process to which journal to submit (Exp(B) = 0.78, 95% CI 0.67–0.91) (Tab. 3). No priority or demographic factors were associated with overall duration between submission and final acceptance (data not displayed).

**Table 3:** Multivariate logistic regression analysis of predictors of acceptance at first-choice journal

	Accepted at first journal (dependent variable)	
Independent variables	*Exp(B)*	*95% CI*
Deciding on journal later	0.77	0.66–0.89
Prioritising editorial reputation	n/s	–
Prioritising speed of dissemination	1.80	1.41–2.28
Prioritising fit	2.11	1.55–2.88
Prioritising impact	0.37	0.28–0.49
Prioritising guidance from others	n/s	–
Prioritising breadth of dissemination	n/s	–
Male gender	n/s	–
Dominant country	n/s	–
Career stage	n/s	–

*n/s* not significant

### Perception of peer review

Over three quarters of participants felt that peer reviewers’ comments enabled them to improve their manuscript either a little (50.9%) or a lot (27.8%). A similar 74.4% of participants felt their final paper was better than the original submitted.

Authors who ultimately had their manuscripts accepted at their first-choice journal had more positive views of the peer reviewer feedback received (mean score = 3.5) than those who did not (mean score = 2.8; t = 18.2, d = 1.5, *p* < 0.001). More interesting, however, is that the factors most associated with whether the author perceived that the feedback enabled them to improve their manuscript were the perceived knowledge of the reviewers (B = 0.16, 95% CI = 0.07–0.24) and the perceived constructiveness of the reviews (B = 0.43, 95% CI = 0.34–0.52). No correlation was seen between perception of review and duration from first rejection to ultimate acceptance for manuscripts that were not accepted at the first journal (r = 0.02, *p* = 0.44). Those who had made substantial changes to their manuscripts before submitting to a second journal had a more positive view of the peer review feedback they had received (mean score = 3.2) compared to those that made cosmetic changes (2.7; t = 3.96, *p* < 0.001).

## Discussion

This study aimed to investigate how HPE scholars choose to which journals to submit their manuscripts, whether or not strategies differ between subgroups of scholars, and what their publication experience was like once a choice was made. The most important priority for authors within our sample was the fit between their manuscript and the journal. This is important because even within this narrow sample of successful authors, prioritising fit more highly was associated with the likelihood of a successful first submission. While all groups rated fit as their top priority, authors from countries that dominate HPE publishing rated editorial reputation, impact, speed of dissemination, and breadth of dissemination as lower priorities than those from non-dominant countries. The likelihood of being published in the first journal to which one submitted was not different between those from dominant and non-dominant countries who did achieve publication, but we have no data on the amount of time and energy spent pursuing journals that are a poor fit by those who were not ultimately successful.

That said, the full list of prioritisation reasons should likely be considered by those who are trying to find their way to success in the field, given that the most important factor is arguably dependent on the personal and professional context and goals of each author. Newcomers will inevitably require guidance in this regard and, as such, it was comforting to see that trainees and junior academics highly prioritised guidance from others, suggesting the value of drawing on the knowledge of those with more experience as a means to achieve publication success (and likely explaining why the odds of being accepted at the first journal to which our respondents submitted were equal in junior and senior participants). We also found that deciding on the journal to which one intends to submit one’s manuscript earlier within the research process was associated with higher odds of acceptance.

Contrary to prior research, the majority of our respondents felt that peer review enabled them to improve their manuscripts [8]. Unsurprisingly, authors who have their manuscripts accepted at the first journal have a more positive perception of the feedback they received from peer reviewers. The aspects of peer review that lead authors to perceive value are the constructiveness of the reviews and the perceived knowledge of the reviewers. Given the oft-bemoaned inconsistency of reviewers, it was notable that consistency was not significantly associated with authors’ perceptions that the review improved their manuscripts. It was concerning that only a minority of authors who were unsuccessful at their first-choice journal made substantial changes to their manuscripts. While all of these manuscripts were ultimately published, one wonders how many more did not make it into the population of accepted articles because the authors did not engage substantively with the feedback received from reviewers.

### Strengths and limitations

This is the first survey of HPE scholars’ motivations and priorities when publishing manuscripts although it builds on previous qualitative research [7]. While previous research had a predominantly senior and North American sample, our study employed a large and highly diverse sample of HPE scholars. Sampling adequacy for the principal component analyses were in the ranges termed ‘middling’ and ‘meritorious’ by Kaiser and Rice [17]. These strengths notwithstanding, our study has several limitations. First, we surveyed only authors whose manuscripts were published. While this enables us to offer guidance based on success stories, it prevents us from knowing if authors of manuscripts that were never accepted adopt different approaches. The manuscripts in the study were also identified through PubMed; as such, our findings may not be representative of those that ultimately ended up having their manuscripts accepted in non-Medline indexed journals, and may exclude articles published in languages other than English. Our use of keyword and MeSH searching may also omit relevant HPE papers. Recent efforts to delineate the field of medical education have highlighted the challenges of identifying a comprehensive approach [18]. We considered the alternative of using articles published within a core set of HPE journals would lead to greater bias than our search strategy.

Second, we have through necessity adopted a cross-sectional design. This makes it difficult to make causal inferences and makes our study susceptible to recall and social desirability biases [19]. Indeed, that 63% of our sample was accepted in the first journal to which they submitted suggests that those who were accepted at their first journal were more likely to respond to the survey relative to the general population of HPE authors (or more likely to claim they sent their manuscript to only one journal). Limitations in autobiographical memory might have been particularly problematic for those respondents who authored multiple papers within the sampling window, but we attempted to minimise that risk by always asking about the most recent paper we uncovered when defining the study population. Surveying the corresponding author may have under-represented any team-based decision processes that informed journal choice. In that regard, it would also have been useful to have more trustworthy information regarding the location and perspectives from which all authors and all journals came to enable deeper analysis of geographic, demographic, and other differences in the patterns observed. Finally, while the number of responses is satisfactory, the response rate was lower than desired. That said, it is in line with the response rates seen in other survey research of HPE authors [13], and our wave analysis suggests very little nonresponse bias.

### Implications for practice

Based on our findings we can offer a number of recommendations to scholars in the field. First, do not be disheartened by a ‘revise’ decision in the way that we have often observed new authors to be; an invitation to revise is an expression of an editor’s interest. Nearly all manuscripts that were published at the ‘first-choice’ journal were accepted only after revisions were made. Not receiving such an offer, however, does not necessarily mean the issues cannot be addressed or that a journal that better ‘fits’ the scope of the project cannot be found. Authors submitted their manuscripts to 1.5 journals on average prior to publication and we suspect that number to be an under-estimate given the direction of bias that is most likely to impact upon these data.

Second, start thinking about the journal to which you would ideally submit early in your research process. In doing so, consider where your manuscript best ‘fits’ in terms of the scope of the journal and matching your work to their readership to maximise your chance of acceptance. Prioritising fit was not associated with an increase in duration between submission and acceptance compared to prioritising speed of dissemination, and the overall delay between manuscript completion and publication is likely to be lowest if the right journal is selected first rather than sending one’s article to a different journal for the wrong reasons.

In determining where one’s manuscript might best ‘fit,’ we suggest authors familiarise themselves with a breadth of journals through reading their articles, editorials, author instructions, and discussing journal selection with experienced HPE colleagues.

## Supplementary Information

Survey

**Table S1** Demographic characteristics of included respondents

**Table S2** Participants’ ratings for motivations for publication items

**Table S3** Summary of principal component analysis of motivations for publication (n = 691)

**Table S4** Participants’ ratings for priorities for journal choice items

**Table S5** Summary of principal component analysis of priorities for journal choice (n = 688)

## References

[1] Sherbino J, Frank JR, Snell L (2014). Defining the key roles and competencies of the clinician-educator of the 21st century: A national mixed-methods study. Acad Med.

[2] Glassick CE (2000). Boyer’s expanded definitions of scholarship, the standards for assessing scholarship, and the elusiveness of the scholarship of teaching. Acad Med.

[3] Hu WCY, Thistlethwaite JE, Weller J, Gallego G, Monteith J, McColl GJ (2015). “It was serendipity”: A qualitative study of academic careers in medical education. Med Educ.

[4] Doja A, Horsley T, Sampson M (2014). Productivity in medical education research: An examination of countries of origin. BMC Med Educ.

[5] Thomas MP (2019). The geographic and topical landscape of medical education research. BMC Med Educ.

[6] Deputy editors of Medical Education (2012). Good advice from the deputy editors of Medical Education. Med Educ.

[7] Ginsburg S, Lynch M, Walsh CM (2018). A fine balance: How authors strategize around journal submission. Acad Med.

[8] Frank E (1994). Authors’ criteria for selecting journals. JAMA.

[9] Sandesh N, Wahrekar S (2017). Choosing the scientific journal for publishing research work: Perceptions of medical and dental researchers. Clujul Med.

[10] Eva KW (2009). The reviewer is always right: Peer review of research in Medical Education. Med Educ.

[11] Calcagno V, Demoinet E, Gollner K, Guidi L, Ruths D, de Mazancourt C (2012). Flows of research manuscripts among scientific journals reveal hidden submission patterns. Science.

[12] Dillman DA, Smyth JD, Christian LM (2014). Internet, phone, mail, and mixed-mode surveys: The tailored design method.

[13] Artino AR, Driessen EW, Maggio LA (2019). Ethical shades of gray: International frequency of scientific misconduct and questionable research practices in health professions education. Acad Med.

[14] Superchi C, González JA, Solà I, Cobo E, Hren D, Boutron I (2019). Tools used to assess the quality of peer review reports: A methodological systematic review. BMC Med Res Methodol.

[15] Phillips AW, Reddy S, Durning SJ (2016). Improving response rates and evaluating nonresponse bias in surveys: AMEE Guide No. 102. Med Teach.

[16] The American Association for Public Opinion Research (2016). Standard definitions: Final dispositions of case codes and outcome rates for surveys.

[17] Kaiser HF, Rice J (1974). Little Jiffy, Mark IV. Educ Psychol Meas.

[18] Maggio LA, Ninkov A, Frank JR, Costello JA, Artino AR (2021). Delineating the field of medical education: Bibliometric research approach(es). Med Educ.

[19] Wang X, Cheng Z (2020). Cross-sectional studies: Strengths, weaknesses, and recommendations. Chest.

