# Supplementary material for: A method for the madness: An international survey of health professions education authors’ journal choice
Source: Perspect Med Educ. 2022 Feb 22;11(3):165–72. doi: 10.1007/s40037-022-00698-9 (PMC9240136; doi:10.1007/s40037-022-00698-9)
Supplement: Supplementary file 1 — Survey [file 40037_2022_698_MOESM1_ESM.pdf]

# Investigating Health Professions Education authors’ journal choice

**Consent Form**

Version 1.0, dated 02/02/2021

**Title of Project**: Investigating Health Professions Education authors' journal choice

**Name and Contact Details of Researcher(s):**

Dr Eliot Rees, School of Medicine, Keele University. e.rees@keele.ac.uk

Professor Kevin Eva, Centre for Health Education Scholarship, University of British Columbia

**REC Reference Number:** KR-210048

Before you consent to participating in the research, please read the **participant information sheet** and then indicate that you agree with each of the statements below in order to continue. If you have any questions or queries before signing the consent form please speak to the researcher.

If you would answer No to any of the questions below, please do not proceed until you have clarified what your participation will mean by contacting the researcher.

**Q1: I have read and understood the research information sheet dated 26/03/2021 (version 03) or the project has been fully explained to me.**

◯ Yes

**Q2: I have been given the opportunity to ask questions about the project and have had these answered satisfactorily.**

◯ Yes

**Q3: I understand that my taking part is voluntary. I also understand that I can discontinue participation at any point up until I have submitted my responses. I do not have to give any reasons for why I no longer want to take part and there will be no adverse consequences if I choose to discontiue the questionnaire.**

◯ Yes

**Q4: I understand that data collected during this research will be processed in accordance with data protection law as explained in the Participant Information Sheet**

◯ Yes

**Q5: I agree to take part in the above research**

◯ Yes

# Eligibility

**Q6: What type of manuscript is '${PaperTitle}'?**

- ◯ Editorial
- ◯ Letter
- ◯ Research article
- ◯ Review article
- ◯ Other article (e.g. commentary, perspective piece, innovation report) - please specify

If you have chosen "other", please specify:

**Q7: Was this paper commissioned?**

◯ Yes ◯ No

# Demographics

*Note: if you have answered/chosen item [1, 2] in question 6, skip the following question*
*Note: if you have answered/chosen item [1] in question 7, skip the following question*

**Q8: How old are you?**

*Note: if you have answered/chosen item [1, 2] in question 6, skip the following question*
*Note: if you have answered/chosen item [1] in question 7, skip the following question*

**Q9: What is your gender identity?**

- ◯ Agender
- ◯ Man
- ◯ Non Binary
- ◯ Trans/ Transgender
- ◯ Woman
- ◯ Prefer not to say
- ◯ Prefer to self describe

If you have chosen "other", please specify:

*Note: if you have answered/chosen item [1, 2] in question 6, skip the following question*
*Note: if you have answered/chosen item [1] in question 7, skip the following question*

**Q10: In what country do you currently work?**

- ○ Afghanistan
- ○ Albania
- ○ Algeria
- ○ Andorra
- ○ Angola
- ○ Antigua & Deps
- ○ Argentina
- ○ Armenia
- ○ Australia
- ○ Austria
- ○ Azerbaijan
- ○ Bahamas
- ○ Bahrain
- ○ Bangladesh
- ○ Barbados
- ○ Belarus
- ○ Belgium
- ○ Belize
- ○ Benin
- ○ Bhutan
- ○ Bolivia
- ○ Bosnia Herzegovina
- ○ Botswana
- ○ Brazil
- ○ Brunei
- ○ Bulgaria
- ○ Burkina
- ○ Burundi
- ○ Cambodia
- ○ Cameroon
- ○ Canada
- ○ Cape Verde
- ○ Central African Rep
- ○ Chad
- ○ Chile
- ○ China
- ○ Colombia
- ○ Comoros
- ○ Congo
- ○ Congo {Democratic Rep}
- ○ Costa Rica
- ○ Croatia
- ○ Cuba
- ○ Cyprus
- ○ Czech Republic
- ○ Denmark
- ○ Djibouti
- ○ Dominica
- ○ Dominican Republic
- ○ East Timor
- ○ Ecuador
- ○ Egypt
- ○ El Salvador
- ○ Equatorial Guinea
- ○ Eritrea
- ○ Estonia
- ○ Ethiopia
- ○ Fiji
- ○ Finland
- ○ France
- ○ Gabon
- ○ Gambia
- ○ Georgia
- ○ Germany
- ○ Ghana
- ○ Greece
- ○ Grenada
- ○ Guatemala
- ○ Guinea
- ○ Guinea-Bissau
- ○ Guyana
- ○ Haiti
- ○ Honduras
- ○ Hungary
- ○ Iceland
- ○ India
- ○ Indonesia
- ○ Iran
- ○ Iraq
- ○ Ireland {Republic}
- ○ Israel
- ○ Italy
- ○ Ivory Coast
- ○ Jamaica
- ○ Japan
- ○ Jordan
- ○ Kazakhstan
- ○ Kenya
- ○ Kiribati
- ○ Korea North
- ○ Korea South
- ○ Kosovo
- ○ Kuwait
- ○ Kyrgyzstan
- ○ Laos
- ○ Latvia
- ○ Lebanon
- ○ Lesotho
- ○ Liberia
- ○ Libya
- ○ Liechtenstein
- ○ Lithuania
- ○ Luxembourg
- ○ Macedonia
- ○ Madagascar
- ○ Malawi
- ○ Malaysia
- ○ Maldives
- ○ Mali
- ○ Malta
- ○ Marshall Islands
- ○ Mauritania
- ○ Mauritius
- ○ Mexico
- ○ Micronesia
- ○ Moldova
- ○ Monaco
- ○ Mongolia
- ○ Montenegro
- ○ Morocco
- ○ Mozambique
- ○ Myanmar, {Burma}
- ○ Namibia
- ○ Nauru
- ○ Nepal
- ○ Netherlands
- ○ New Zealand
- ○ Nicaragua
- ○ Niger
- ○ Nigeria
- ○ Norway
- ○ Oman
- ○ Pakistan
- ○ Palau
- ○ Panama
- ○ Papua New Guinea
- ○ Paraguay
- ○ Peru
- ○ Philippines
- ○ Poland
- ○ Portugal
- ○ Qatar
- ○ Romania
- ○ Russian Federation

- Rwanda
- Saint Vincent & the Grenadines
- Samoa
- San Marino
- Sao Tome & Principe
- Saudi Arabia
- Senegal
- Serbia
- Seychelles
- Sierra Leone
- Singapore
- Slovakia
- Slovenia
- Solomon Islands
- Somalia
- South Africa
- South Sudan
- Spain
- Sri Lanka
- St Kitts & Nevis
- St Lucia
- Sudan
- Suriname
- Swaziland
- Sweden
- Switzerland
- Syria
- Taiwan
- Tajikistan
- Tanzania
- Thailand
- Togo
- Tonga
- Trinidad & Tobago
- Tunisia
- Turkey
- Turkmenistan
- Tuvalu
- Uganda
- Ukraine
- United Arab Emirates
- United Kingdom
- United States
- Uruguay
- Uzbekistan
- Vanuatu
- Vatican City
- Venezuela
- Vietnam
- Yemen
- Zambia
- Zimbabwe

*Note: if you have answered/chosen item [1, 2] in question 6, skip the following question*
*Note: if you have answered/chosen item [1] in question 7, skip the following question*

**Q11: Which degree(s) do you hold?(Please select all that apply)**

- [ ] Bachelor's degree (BA, BSc, etc.)
- [ ] Master's degree (MA, MSc, MPH, MBA etc.)
- [ ] Primary Medical Qualification (MBChB, MBBS, MD, etc.)
- [ ] Doctoral degree (PhD, EdD, DPhil, MD by research, etc.)
- [ ] None
- [ ] Other

If you have chosen "other", please specify:

*Note: if you have answered/chosen item [1, 2] in question 6, skip the following question*
*Note: if you have answered/chosen item [1] in question 7, skip the following question*

**Q12: For the highest degree you selected above, what is your primary area of study?(please select one)**

- Basic Science
- Clinical Science
- Social Science / Education
- Humanities
- Other

If you have chosen "other", please specify:

# Professional background

Please answer all of these questions to indicate your professional role at the time that you wrote ' **${PaperTitle}'**

*Note: if you have answered/chosen item [1, 2] in question 6, skip the following question*
*Note: if you have answered/chosen item [1] in question 7, skip the following question*

**Q13: At the time of writing this paper, what was your academic rank or position title?**

- ◯ Undergraduate / Health Professions Student
- ◯ Resident / Postgraduate Clinical Trainee
- ◯ Master's Student
- ◯ PhD Student (or equivalent)
- ◯ Teaching Fellow / Clinical Teaching Fellow
- ◯ Postdoctoral Research Associate / Research Fellow
- ◯ Instructor / Sessional Tutor / Clinical Tutor
- ◯ Lecturer / Assistant Professor / Clinical Lecturer
- ◯ Senior Lecturer / Associate Professor / Senior Clinical Lecturer
- ◯ Reader
- ◯ Professor
- ◯ Emeritus Professor
- ◯ Not applicable
- ◯ Other

If you have chosen "other", please specify:

*Note: if you have answered/chosen item [1, 2] in question 6, skip the following question*
*Note: if you have answered/chosen item [1] in question 7, skip the following question*

**Q14: What are your work roles?(please select all that apply)**

- ☐ Clinician
- ☐ Administrator or Program Director (e.g. admissions lead, curriculum lead, assessment lead, associate dean)
- ☐ Teacher (clinical or classroom setting)
- ☐ Researcher
- ☐ Other

If you have chosen "other", please specify:

*Note: if you have answered/chosen item [1, 2] in question 6, skip the following question*
*Note: if you have answered/chosen item [1] in question 7, skip the following question*

**Q15: Which of the following best describes the context in which you work?(please select all that apply)**

☐ Undergraduate Health Professions Education ☐ Graduate Health Professions Education
☐ Continuing Health Professions Education

*Note: if you have answered/chosen item [1, 2] in question 6, skip the following question*
*Note: if you have answered/chosen item [1] in question 7, skip the following question*

**Q16: In a typical work week, approximately what percentage of your work time do you spend on health professions education research activities, including writing up your research?**

[ ] %

*Note: if you have answered/chosen item [1, 2] in question 6, skip the following question*
*Note: if you have answered/chosen item [1] in question 7, skip the following question*

**Q17: How many years had you been involved in health professions education (in any capacity)?**

[ ] years

*Note: if you have answered/chosen item [1, 2] in question 6, skip the following question*
*Note: if you have answered/chosen item [1] in question 7, skip the following question*

**Q18: How many years had you been involved in conducting research in health professions education?**

[ ] years

*Note: if you have answered/chosen item [1, 2] in question 6, skip the following question*
*Note: if you have answered/chosen item [1] in question 7, skip the following question*

**Q19: In thinking about your primary research activities, with which label do you most identify?(Please select one)**

○ I am a quantitative researcher ○ I am a qualitative researcher ○ I am a mixed methods researcher

*Note: if you have answered/chosen item [1, 2] in question 6, skip the following question*
*Note: if you have answered/chosen item [1] in question 7, skip the following question*

**Q20: How many publications do you have in peer reviewed journals?**
**(This can include research papers, reviews, commentaries, letters or other articles in peer reviewed journals)**

[ ]

*Note: if you have answered/chosen item [1, 2] in question 6, skip the following question*
*Note: if you have answered/chosen item [1] in question 7, skip the following question*

**Q21: What proportion of your scholarly output relates to health professions education?**

○ Less than 25% ○ 25% to 49% ○ 50% to 74% ○ 75% to 100%

*Note: if you have answered/chosen item [1, 2] in question 6, skip the following question*
*Note: if you have answered/chosen item [1] in question 7, skip the following question*

**Q22: For what proportion of your scholarly work are you first author?**

○ Less than 25% ○ 25% to 49% ○ 50% to 74% ○ 75% to 100%

*Note: if you have answered/chosen item [1, 2] in question 6, skip the following question*
*Note: if you have answered/chosen item [1] in question 7, skip the following question*

**Q23: In the past 12 months, for approximately how many health professions education articles have you acted as a peer reviewer?**

○ 0 ○ 1 ○ 2-5 ○ 6-10 ○ More than 10

*Note: if you have answered/chosen item [1, 2] in question 6, skip the following question*
*Note: if you have answered/chosen item [1] in question 7, skip the following question*

**Q24: Do you have an editorial role at a health professions education journal?**

○ Yes ○ No

## Motivations for publication

*Note: if you have answered/chosen item [1, 2] in question 6, skip the following question*
*Note: if you have answered/chosen item [1] in question 7, skip the following question*

**Q25: In deciding where to submit your paper '${PaperTitle}', to what extent did each of the following goals influence your decision?**

| | Not important | Slightly important | Moderately important | Very important | Essential |
|---|---|---|---|---|---|
| To advance knowledge in the field | ○ | ○ | ○ | ○ | ○ |
| To communicate to others in the field | ○ | ○ | ○ | ○ | ○ |
| To enjoy the thrill of seeing my work in print | ○ | ○ | ○ | ○ | ○ |
| To get feedback from peer reviewers | ○ | ○ | ○ | ○ | ○ |
| To enable networking with others in the field | ○ | ○ | ○ | ○ | ○ |

| | | | | | |
|---|---|---|---|---|---|
| To assist with winning grants and research support | ◯ | ◯ | ◯ | ◯ | ◯ |
| To act as a catalyst for attracting high quality staff and students | ◯ | ◯ | ◯ | ◯ | ◯ |
| To enable promotion or other type of career advancement | ◯ | ◯ | ◯ | ◯ | ◯ |
| To develop my national / international reputation | ◯ | ◯ | ◯ | ◯ | ◯ |
| To support career development of my co-authors | ◯ | ◯ | ◯ | ◯ | ◯ |

## First choice journal

*Note: if you have answered/chosen item [1, 2] in question 6, skip the following question*
*Note: if you have answered/chosen item [1] in question 7, skip the following question*

**Q26: What was the first journal to which you submitted this manuscript?**

*Note: if you have answered/chosen item [1, 2] in question 6, skip the following question*
*Note: if you have answered/chosen item [1] in question 7, skip the following question*

**Q27: At what stage in your process did you decide this was the right journal to which to submit?**

- ◯ Before designing the project
- ◯ While conducting the project
- ◯ After completing data analysis but before writing the manuscript
- ◯ While writing the manuscript
- ◯ After manuscript completion

## Priorities for first choice journal

*Note: if you have answered/chosen item [1, 2] in question 6, skip the following question*
*Note: if you have answered/chosen item [1] in question 7, skip the following question*

**Q28: When choosing to submit your manucript '${PaperTitle}' to ${1stJournal}, how important, if at all, were the following factors?**

| | Not important | Slightly important | Moderately important | Very important | Essential |
|---|---|---|---|---|---|
| Ability to publish open access | ◯ | ◯ | ◯ | ◯ | ◯ |

| | | | | | |
|---|---|---|---|---|---|
| Acceptance rate | ○ | ○ | ○ | ○ | ○ |
| Attention the journal gets in the press | ○ | ○ | ○ | ○ | ○ |
| Databases in which the journal is indexed | ○ | ○ | ○ | ○ | ○ |
| Familiarity with the journal | ○ | ○ | ○ | ○ | ○ |
| Focus of the journal | ○ | ○ | ○ | ○ | ○ |
| Geographic distribution of its readership | ○ | ○ | ○ | ○ | ○ |
| Impact factor | ○ | ○ | ○ | ○ | ○ |
| Instruction from Department Head/Supervisor to submit there | ○ | ○ | ○ | ○ | ○ |
| Journal’s activity on social media | ○ | ○ | ○ | ○ | ○ |
| Journal’s link with a society or organisation | ○ | ○ | ○ | ○ | ○ |
| Manuscript types the journal accepts | ○ | ○ | ○ | ○ | ○ |
| Match between the journal's readership and the audience I hope to reach | ○ | ○ | ○ | ○ | ○ |
| Reputation for making decisions on manuscripts quickly | ○ | ○ | ○ | ○ | ○ |
| Reputation for publishing rigorous research | ○ | ○ | ○ | ○ | ○ |
| Reputation for useful feedback during peer review | ○ | ○ | ○ | ○ | ○ |
| Reputation of the editor | ○ | ○ | ○ | ○ | ○ |
| Reputation of the editorial board | ○ | ○ | ○ | ○ | ○ |

| | | | | | |
|---|---|---|---|---|---|
| Size of print circulation | ○ | ○ | ○ | ○ | ○ |
| Suggestions from colleagues | ○ | ○ | ○ | ○ | ○ |
| Time taken to publish accepted manuscripts | ○ | ○ | ○ | ○ | ○ |

*Note: if you have answered/chosen item [1, 2] in question 6, skip the following question*
*Note: if you have answered/chosen item [1] in question 7, skip the following question*

**Q29: Please offer any other factors that you think to have been important and/or clarify your responses as necessary**

*Note: if you have answered/chosen item [1, 2] in question 6, skip the following question*
*Note: if you have answered/chosen item [1] in question 7, skip the following question*

**Q30: Before you submitted this manuscript, how likely did you think it was to be accepted in ${1stJournal}?**

○ Very unlikely ○ Somewhat unlikely ○ Neither likely nor unlikely
○ Somewhat likely ○ Very likely

*Note: if you have answered/chosen item [1, 2] in question 6, skip the following question*
*Note: if you have answered/chosen item [1] in question 7, skip the following question*

**Q31: Have you submitted any previous papers to ${1stJournal}?**

○ Yes, and at least one has been accepted/published ○ Yes, but none have been accepted
○ No

# Outcomes from first choice journal

*Note: if you have answered/chosen item [1, 2] in question 6, skip the following question*
*Note: if you have answered/chosen item [1] in question 7, skip the following question*

**Q32: What was the initial decision for this manuscript?**

- ◯ Rejected without peer review
- ◯ Rejected after peer review
- ◯ Major revisions
- ◯ Minor revisions
- ◯ Accepted without revisions

*Note: if you have answered/chosen item [1, 2] in question 6, skip the following question*
*Note: if you have answered/chosen item [1] in question 7, skip the following question*

**Q33: Was the manuscript eventually accepted at this journal?**

- ◯ Yes
- ◯ No

*Note: if you have answered/chosen item [1, 2] in question 6, skip the following question*
*Note: if you have answered/chosen item [1] in question 7, skip the following question*

**Q34: How many rounds of revisions were completed prior to acceptance?**

- ◯ N/A
- ◯ 1
- ◯ 2
- ◯ 3
- ◯ 4
- ◯ 5 or more

# Peer review from first journal

Please think back to the feedback you received from peer reviewers and/or the editor after submitting **'${PaperTitle}'** to ***${1stJournal}***.

*Note: if you have answered/chosen item [1, 2] in question 6, skip the following question*
*Note: if you have answered/chosen item [1] in question 7, skip the following question*

**Q35: How clear or unclear was the feedback?**

- ◯ Clear
- ◯ Somewhat clear
- ◯ Somewhat unclear
- ◯ Unclear

*Note: if you have answered/chosen item [1, 2] in question 6, skip the following question*
*Note: if you have answered/chosen item [1] in question 7, skip the following question*

**Q36: How constructive or unhelpful was the feedback?**

- ◯ Constructive
- ◯ Somewhat constructive
- ◯ Somewhat unhelpful
- ◯ Unhelpful

*Note: if you have answered/chosen item [1, 2] in question 6, skip the following question*
*Note: if you have answered/chosen item [1] in question 7, skip the following question*

**Q37: How appropriately detailed was the feedback?**

- Appropriately detailed
- Somewhat appropriately detailed
- Somewhat inappropriately detailed
- Inappropriately detailed

*Note: if you have answered/chosen item [1, 2] in question 6, skip the following question*
*Note: if you have answered/chosen item [1] in question 7, skip the following question*

**Q38: How fair or unfair was the feedback you received?**

- Fair
- Somewhat fair
- Somewhat unfair
- Unfair

*Note: if you have answered/chosen item [1, 2] in question 6, skip the following question*
*Note: if you have answered/chosen item [1] in question 7, skip the following question*

**Q39: How knowledgeable or non-expert did you perceive the reviewers to be?**

- Knowledgeable
- Somewhat knowledgeable
- Somewhat non-expert
- Non-expert

*Note: if you have answered/chosen item [1, 2] in question 6, skip the following question*
*Note: if you have answered/chosen item [1] in question 7, skip the following question*

**Q40: Overall, how courteous or harsh did you perceive the tone to be?**

- Courteous
- Somewhat courteous
- Somewhat harsh
- Harsh

*Note: if you have answered/chosen item [1, 2] in question 6, skip the following question*
*Note: if you have answered/chosen item [1] in question 7, skip the following question*

**Q41: How consistent or conflicting was the feedback from different reviewers?**

- Consistent amongst reviewers
- Somewhat consistent amongst reviewers
- Somewhat conflicting amongst reviewers
- Conflicting amongst reviewers

*Note: if you have answered/chosen item [1, 2] in question 6, skip the following question*
*Note: if you have answered/chosen item [1] in question 7, skip the following question*

**Q42: Overall, to what extent do you think this feedback enabled you to improve your manuscript?**

- Improve the manuscript a lot
- Improve the manuscript a little
- Neither improve nor worsen the manuscript
- Worsen the manuscript a little
- Worsen the manuscript a lot

## Second choice journal

*Note: if you have answered/chosen item [1, 2] in question 6, skip the following question*
*Note: if you have answered/chosen item [1] in question 7, skip the following question*
*Note: if you have answered/chosen item [5] in question 32, skip the following question*
*Note: if you have answered/chosen item [1] in question 33, skip the following question*

**Q43: What was the second journal to which you submitted this manuscript?**

| |
|---|
| |

*Note: if you have answered/chosen item [1, 2] in question 6, skip the following question*
*Note: if you have answered/chosen item [1] in question 7, skip the following question*
*Note: if you have answered/chosen item [5] in question 32, skip the following question*
*Note: if you have answered/chosen item [1] in question 33, skip the following question*

**Q44: What were the top three factors in determining your second choice?(please select 3 options)**

- ☐ Ability to publish open access
- ☐ Acceptance rate
- ☐ Attention the journal gets in the press
- ☐ Databases in which the journal is indexed
- ☐ Familiarity with the journal
- ☐ Focus of the journal
- ☐ Geographic distribution of its readership
- ☐ Impact factor
- ☐ Instruction from Department Head/Supervisor to submit there
- ☐ Journal’s activity on social media
- ☐ Journal’s link with a society or organisation
- ☐ Manuscript types the journal accepts
- ☐ Reputation for making decisions on manuscripts quickly
- ☐ Reputation for publishing rigorous research
- ☐ Reputation for useful feedback during peer review
- ☐ Reputation of the editor
- ☐ Reputation of the editorial board
- ☐ Size of print circulation
- ☐ Suggestions from colleagues
- ☐ Time taken to publish accepted manuscripts

*Note: if you have answered/chosen item [1, 2] in question 6, skip the following question*
*Note: if you have answered/chosen item [1] in question 7, skip the following question*
*Note: if you have answered/chosen item [5] in question 32, skip the following question*
*Note: if you have answered/chosen item [1] in question 33, skip the following question*

**Q45: Prior to submitting to the second journal, what actions did you take?(please select one)**

- ○ Just re-submitted the original manuscript
- ○ Changed the formatting to meet the new journal’s requirements
- ○ Tweaked the wording
- ○ Made changes to the reporting without significant changes to content
- ○ Made substantial changes to the manuscript (e.g. conceptually reframed the article or added substantially to the analysis)

*Note: if you have answered/chosen item [1, 2] in question 6, skip the following question*
*Note: if you have answered/chosen item [1] in question 7, skip the following question*
*Note: if you have answered/chosen item [5] in question 32, skip the following question*
*Note: if you have answered/chosen item [1] in question 33, skip the following question*

**Q46: To which sections of the manuscript did you make changes?(please select all that apply)**

☐ Title ☐ Abstract ☐ Introduction ☐ Methods ☐ Results ☐ Discussion
☐ References

*Note: if you have answered/chosen item [1, 2] in question 6, skip the following question*
*Note: if you have answered/chosen item [1] in question 7, skip the following question*
*Note: if you have answered/chosen item [5] in question 32, skip the following question*
*Note: if you have answered/chosen item [1] in question 33, skip the following question*

**Q47: Why did you make these changes?(please select all that apply)**

☐ In response to the original reviewers' comments ☐ To better align with the new journal's focus

*Note: if you have answered/chosen item [1, 2] in question 6, skip the following question*
*Note: if you have answered/chosen item [1] in question 7, skip the following question*
*Note: if you have answered/chosen item [5] in question 32, skip the following question*
*Note: if you have answered/chosen item [1] in question 33, skip the following question*

**Q48: Please offer any other comments you think would help to clarify your responses as necessary**

# Outcomes from second choice journal

*Note: if you have answered/chosen item [1, 2] in question 6, skip the following question*
*Note: if you have answered/chosen item [1] in question 7, skip the following question*
*Note: if you have answered/chosen item [5] in question 32, skip the following question*
*Note: if you have answered/chosen item [1] in question 33, skip the following question*

**Q49: What was the initial decision for this manuscript?**

○ Rejected without peer review ○ Rejected after peer review ○ Major revisions
○ Minor revisions ○ Accepted without revisions

*Note: if you have answered/chosen item [1, 2] in question 6, skip the following question*
*Note: if you have answered/chosen item [1] in question 7, skip the following question*
*Note: if you have answered/chosen item [5] in question 32, skip the following question*
*Note: if you have answered/chosen item [1] in question 33, skip the following question*

**Q50: Was the paper eventually accepted at this journal?**

○ Yes ○ No

*Note: if you have answered/chosen item [1, 2] in question 6, skip the following question*
*Note: if you have answered/chosen item [1] in question 7, skip the following question*
*Note: if you have answered/chosen item [5] in question 32, skip the following question*
*Note: if you have answered/chosen item [1] in question 33, skip the following question*

**Q51: If so, how many revisions were completed prior to acceptance?**

○ N/A ○ 1 ○ 2 ○ 3 ○ 4 ○ 5 or more

*Note: if you have answered/chosen item [1, 2] in question 6, skip the following question*
*Note: if you have answered/chosen item [1] in question 7, skip the following question*
*Note: if you have answered/chosen item [5] in question 32, skip the following question*
*Note: if you have answered/chosen item [1] in question 33, skip the following question*

**Q52: If not, to how many other journals did you submit before you were eventually published?**

[ ] journals

*Note: if you have answered/chosen item [1, 2] in question 6, skip the following question*
*Note: if you have answered/chosen item [1] in question 7, skip the following question*
*Note: if you have answered/chosen item [5] in question 32, skip the following question*
*Note: if you have answered/chosen item [1] in question 33, skip the following question*

**Q53: Why do you think the eventual journal that accepted the article did so, whereas the first one did not?**

*Note: if you have answered/chosen item [1, 2] in question 6, skip the following question*
*Note: if you have answered/chosen item [1] in question 7, skip the following question*

**Q54: How satisfied or disatisfied were you with the eventual home for this manuscript?**

◯ Very satisfied ◯ Somewhat satisfied ◯ Neither satisfied nor dissatisfied
◯ Dissatisfied ◯ Very dissatisfied

Why?

*Note: if you have answered/chosen item [1, 2] in question 6, skip the following question*
*Note: if you have answered/chosen item [1] in question 7, skip the following question*
*Note: if you have answered/chosen item [5] in question 32, skip the following question*

**Q55: In the end do you think the paper to be better, the same quality, or worse relative to when it was first submitted?**

◯ Better ◯ The same quality ◯ Worse

## Final decision

*Note: if you have answered/chosen item [1, 2] in question 6, skip the following question*
*Note: if you have answered/chosen item [1] in question 7, skip the following question*

**Q56: Please estimate how many weeks elapsed between submitting the manuscript to ${1stJournal} and receiving an initial decision.**

Weeks

*Note: if you have answered/chosen item [1, 2] in question 6, skip the following question*
*Note: if you have answered/chosen item [1] in question 7, skip the following question*
*Note: if you have answered/chosen item [5] in question 32, skip the following question*
*Note: if you have answered/chosen item [1] in question 33, skip the following question*

**Q57: Please estimate how many weeks elapsed between submitting the manuscript to ${2ndJournal} and receiving an initial decision.**

Weeks

*Note: if you have answered/chosen item [1, 2] in question 6, skip the following question*
*Note: if you have answered/chosen item [1] in question 7, skip the following question*

**Q58: Please estimate how many weeks elapsed between initially submitting the manuscript to ${1stJournal} and it finally being accepted.**

Weeks

*Note: if you have answered/chosen item [1, 2] in question 6, skip the following question*
*Note: if you have answered/chosen item [1] in question 7, skip the following question*

**Q59: What else do you think is important for someone to know if they are trying to understand the journey this paper went through prior to achieving publication?**
