# Supplementary material for: A method for the madness: An international survey of health professions education authors’ journal choice
Source: Perspect Med Educ. 2022 Feb 22;11(3):165–72. doi: 10.1007/s40037-022-00698-9 (PMC9240136; doi:10.1007/s40037-022-00698-9)
Supplement: Supplementary file 2 — Table S1 Demographic characteristics of included respondents [file 40037_2022_698_MOESM2_ESM.docx]

**Table S1** Demographic characteristics of included respondents

| **Demographic characteristic** | **No.** | **%** |
| --- | --- | --- |
| Gender identity |  |  |
| Male | 326 | 47.2 |
| Female | 354 | 51.2 |
| Trans/Transgender | 1 | 0.1 |
| Prefer not to say | 10 | 1.4 |
| Continent |  |  |
| Africa | 23 | 3.3 |
| Asia | 93 | 13.5 |
| Australia | 40 | 5.8 |
| Europe | 167 | 24.2 |
| North America | 348 | 50.4 |
| South America | 20 | 2.9 |
| Work role* |  |  |
| Clinician | 384 | 55.7 |
| Administrator / program director | 288 | 41.8 |
| Teacher | 437 | 63.4 |
| Researcher | 490 | 71.1 |
| Other | 50 | 7.3 |
| Not reported | 2 | 0.3 |
| Academic Rank |  |  |
| Undergraduate / Health Professions student | 20 | 2.9 |
| Resident / postgraduate clinical trainee | 33 | 4.9 |
| Master’s student | 9 | 1.3 |
| PhD Student (or equivalent) | 68 | 10.0 |
| Teaching Fellow | 13 | 1.9 |
| Postdoctoral research associate | 24 | 3.5 |
| Instructor / sessional tutor / clinical tutor | 30 | 4.4 |
| Lecturer / assistant professor | 171 | 25.2 |
| Senior lecturer / associate professor | 160 | 23.6 |
| Reader | 4 | 0.6 |
| Professor | 145 | 21.4 |
| Emeritus professor | 2 | 0.3 |
| Not applicable | 12 | 1.7 |
| Primary research activity |  |  |
| Qualitative | 129 | 18.7 |
| Quantitative | 178 | 25.8 |
| Mixed methods | 380 | 55.0 |
| Not reported | 4 | 0.6 |
| Proportion of scholarly output relating to HPE |  |  |
| <25% | 208 | 30.2 |
| 25-49% | 104 | 15.1 |
| 50-74% | 104 | 15.1 |
| 75-100% | 272 | 39.5 |
| Proportion of scholarly output as first author |  |  |
| <25% | 112 | 16.3 |
| 25-49% | 245 | 35.6 |
| 50-74% | 201 | 29.2 |
| 75-100% | 131 | 19.0 |
| Number of HPE articles participant peer reviewed in preceding year. |  |  |
| 0 | 117 | 17.0 |
| 1 | 111 | 16.1 |
| 2-5 | 257 | 37.2 |
| 6-10 | 123 | 17.8 |
| >10 | 82 | 11.9 |
| Editorial role at HPE journal |  |  |
| Currently held | 147 | 21.3 |
| Not currently held | 544 | 78.7 |

* Participants were able to select multiple work roles and, hence, the total surpasses 100%.
