# Supplementary material for: A method for the madness: An international survey of health professions education authors’ journal choice
Source: Perspect Med Educ. 2022 Feb 22;11(3):165–72. doi: 10.1007/s40037-022-00698-9 (PMC9240136; doi:10.1007/s40037-022-00698-9)
Supplement: Supplementary file 3 — Table S2 Participants’ ratings for motivations for publication items [file 40037_2022_698_MOESM3_ESM.docx]

**Table S2** Participants’ ratings for motivations for publication items

| **Motivation** | **Not important** | **Slightly important** | **Moderately important** | **Very important** | **Essential** | **n** | **Mean** | **SD** |
| --- | --- | --- | --- | --- | --- | --- | --- | --- |
| To communicate to others in the field | 0.3 | 1.0 | 10.9 | 47.7 | 40.1 | 690 | **4.26** | **.71** |
| To advance knowledge in the field | 0.4 | 2.9 | 14.6 | 44.6 | 37.5 | 691 | **4.16** | **.81** |
| To develop my national / international reputation | 8.1 | 15.8 | 32.9 | 29.6 | 13.6 | 690 | **3.25** | **1.12** |
| To support career development of my co-authors | 13.1 | 14.7 | 25.9 | 28.5 | 17.8 | 6.87 | **3.23** | **1.27** |
| To get feedback from peer reviewers | 8.3 | 21.7 | 33.8 | 25.9 | 10.3 | 690 | **3.08** | **1.10** |
| To enable networking with others in the field | 9.6 | 19.9 | 32.2 | 29.5 | 8.7 | 687 | **3.08** | **1.11** |
| To enable promotion or other type of career advancement | 17.1 | 18.4 | 25.1 | 24.9 | 14.5 | 690 | **3.01** | **1.30** |
| To enjoy the thrill of seeing my work in print | 14.6 | 27.7 | 28.8 | 21.3 | 7.5 | 690 | **2.79** | **1.16** |
| To assist with winning grants and research support | 35.7 | 27.5 | 20.7 | 11.9 | 4.2 | 691 | **2.21** | **1.17** |
| To act as a catalyst for attracting high quality staff and students | 40.2 | 25.4 | 21.0 | 9.7 | 3.6 | 689 | **2.21** | **1.15** |
