# Supplementary material for: A method for the madness: An international survey of health professions education authors’ journal choice
Source: Perspect Med Educ. 2022 Feb 22;11(3):165–72. doi: 10.1007/s40037-022-00698-9 (PMC9240136; doi:10.1007/s40037-022-00698-9)
Supplement: Supplementary file 4 — Table S3 Summary of principal component analysis of motivations for publication (n = 691) [file 40037_2022_698_MOESM4_ESM.docx]

**Table S3** Summary of principal component analysis of motivations for publication (n=691)

|  |  | **Rotated factor loadings** | | |
| --- | --- | --- | --- | --- |
|  | **Item** | **Personal promotion** | **Advancing the field’s knowledge** | **Capacity building** |
| Factor 1 | To enjoy the thrill of seeing my work in print | **.807** |  |  |
|  | To enable promotion or other type of career advancement | **.740** |  |  |
|  | To develop my national / international reputation | **.673** |  |  |
|  | To get feedback from peer reviewers | .446 |  |  |
|  | To enable networking with others in the field | .362 |  | .320 |
| Factor 2 | To communicate with others in the field |  | **.890** |  |
|  | To advance knowledge in the field |  | **.861** |  |
| Factor 3 | To support career development of my co-authors |  |  | .**855** |
|  | To act as a catalyst for attracting high quality staff and students |  |  | **.663** |
|  | To assist with winning grants and research support | .314 |  | **.576** |
|  | Eigenvalues | 3.37 | 1.52 | 1.00 |
|  | % of variance | 33.75 | 15.15 | 10.01 |
|  | α | .72 | .74 | .62 |

Factor loadings over 0.5 appear in bold. Only factor loading greater than .3 presented.
