# Supplementary material for: A method for the madness: An international survey of health professions education authors’ journal choice
Source: Perspect Med Educ. 2022 Feb 22;11(3):165–72. doi: 10.1007/s40037-022-00698-9 (PMC9240136; doi:10.1007/s40037-022-00698-9)
Supplement: Supplementary file 5 — Table S4 Participants’ ratings for priorities for journal choice items [file 40037_2022_698_MOESM5_ESM.docx]

**Table S4** Participants’ ratings for priorities for journal choice items

| **Priority** | **Not important** | **Slightly important** | **Moderately important** | **Very important** | **Essential** | **n** | **Mean** | **SD** |
| --- | --- | --- | --- | --- | --- | --- | --- | --- |
| Match between the journal's readership and the audience I hope to reach | 1.0 | 3.9 | 15.7 | 39.8 | 39.9 | 668 | **4.13** | **0.89** |
| Focus of the journal | 0.3 | 3.9 | 14.8 | 45.1 | 35.9 | 669 | **4.12** | **0.82** |
| Familiarity with the journal | 1.8 | 9.4 | 25.8 | 47.2 | 15.8 | 670 | **3.66** | **0.92** |
| Manuscript types the journal accepts | 5.2 | 8.4 | 25.3 | 40.2 | 20.9 | 669 | **3.63** | **1.07** |
| Reputation for publishing rigorous research | 5.8 | 8.1 | 23.23 | 42.7 | 20.2 | 668 | **3.63** | **1.07** |
| Databases in which the journal is indexed | 10.1 | 10.1 | 24.9 | 30.7 | 24.0 | 670 | **3.48** | **1.24** |
| Impact factor | 5.8 | 12.7 | 28.3 | 35.1 | 18.1 | 669 | **3.47** | **1.10** |
| Reputation for useful feedback during peer review | 13.7 | 20.0 | 29.8 | 26.3 | 10.2 | 665 | **2.99** | **1.19** |
| Geographic distribution of its readership | 16.9 | 18.3 | 27.3 | 28.0 | 9.4 | 667 | **2.95** | **1.23** |
| Reputation for making decisions on manuscripts quickly | 19.3 | 21.1 | 29.7 | 22.2 | 7.6 | 667 | **2.78** | **1.21** |
| Attention the journal gets in the press | 24.7 | 18.4 | 26.5 | 24.7 | 5.8 | 669 | **2.69** | **1.25** |
| Time taken to publish accepted manuscripts | 24.3 | 22.0 | 28.9 | 19.2 | 5.5 | 667 | **2.60** | **1.20** |
| Reputation of the editor | 28.4 | 25.4 | 24.3 | 15.8 | 6.2 | 666 | **2.46** | **1.22** |
| Acceptance rate | 27.7 | 21.9 | 32.3 | 13.6 | 4.5 | 671 | **2.45** | **1.16** |
| Suggestions from colleagues | 31.0 | 22.0 | 24.4 | 18.3 | 4.2 | 667 | **2.43** | **1.22** |
| Ability to publish open access | 39.8 | 16.3 | 19.3 | 17.4 | 7.1 | 673 | **2.36** | **1.34** |
| Reputation of the editorial board | 32.1 | 26.4 | 23.5 | 13.4 | 4.5 | 663 | **2.32** | **1.18** |
| Journal’s link with a society or organisation | 39.8 | 20.8 | 16.6 | 17.4 | 5.4 | 668 | **2.28** | **1.29** |
| Size of print circulation | 43.8 | 25.3 | 18.1 | 10.6 | 2.1 | 667 | **2.02** | **1.11** |
| Instruction from Department Head/Supervisor to submit there | 65.4 | 11.9 | 10.2 | 8.6 | 3.9 | 665 | **1.74** | **1.18** |
| Journal’s activity on social media | 59.1 | 23.7 | 12.9 | 3.9 | 0.4 | 667 | **1.63** | **0.88** |
