# Supplementary material for: A method for the madness: An international survey of health professions education authors’ journal choice
Source: Perspect Med Educ. 2022 Feb 22;11(3):165–72. doi: 10.1007/s40037-022-00698-9 (PMC9240136; doi:10.1007/s40037-022-00698-9)
Supplement: Supplementary file 6 — Table S5 Summary of principal component analysis of priorities for journal choice (n = 688) [file 40037_2022_698_MOESM6_ESM.docx]

**Table S5** Summary of principal component analysis of priorities for journal choice (n=688)

|  | **Rotated factor loadings** | | | | | |
| --- | --- | --- | --- | --- | --- | --- |
| **Item** | **Editorial reputation** | **Fit** | **Guidance from others** | **Speed of dissemination** | **Impact** | **Breadth of dissemination** |
| Reputation of the editor | **.887** |  |  |  |  |  |
| Reputation of the editorial board | **.887** |  |  |  |  |  |
| Reputation for useful feedback during peer review | **.719** |  |  |  |  |  |
| Focus of the journal |  | **.797** |  |  |  |  |
| Match between the journal’s readership and the audience I hope to reach |  | **.728** |  |  |  |  |
| Manuscript types the journal accepts |  | **.658** |  |  |  |  |
| Familiarity with the journal |  | **.523** |  |  |  |  |
| Instruction from department head / supervisor to submit there |  |  | **.853** |  |  |  |
| Suggestions from colleagues |  |  | **.784** |  |  |  |
| Acceptance rate |  |  |  | **.804** |  |  |
| Ability to publish open access |  |  |  | **.686** |  |  |
| Time taken to publish accepted manuscripts | .400 |  |  | .483 |  |  |
| Reputation for making decisions on manuscripts quickly | .429 |  |  | .483 |  |  |
| Impact factor |  |  |  |  | **.785** |  |
| Attention the journal gets in the press |  |  |  |  | **.654** |  |
| Reputation for publishing rigorous research | .482 |  |  |  | **.504** |  |
| Databases in which the journal is indexed |  |  |  | .429 | .472 |  |
| Journal’s link with a society or organisation |  |  |  |  |  | **.689** |
| Geographic distribution of its readership |  |  |  |  | .319 | .479 |
| Journal’s activity on social media |  |  | .357 |  |  | **.**431 |
| Size of print circulation | .390 | - |  | - | .213 | .429 |
| Eigenvalues | 5.79 | 1.98 | 1.46 | 1.36 | 1.25 | 1.15 |
| % of variance | 27.59 | 9.42 | 6.97 | 6.47 | 5.94 | 5.46 |
| α | .87 | .64 | .61 | .73 | .71 | .60 |

Factor loadings over 0.5 appear in bold. Only factor loading greater than 0.3 presented.
